# Supplementary material for: Stacking sequence and interlayer coupling in few-layer graphene revealed by in situ imaging
Source: Nat Commun. 2016 Oct 19;7:13256. doi: 10.1038/ncomms13256 (PMC5075831; doi:10.1038/ncomms13256)
Supplement: Supplementary Information — Supplementary Figures 1-5, Supplementary Notes 1-4 and Supplementary References [file ncomms13256-s1.pdf]

Supplementary Figures

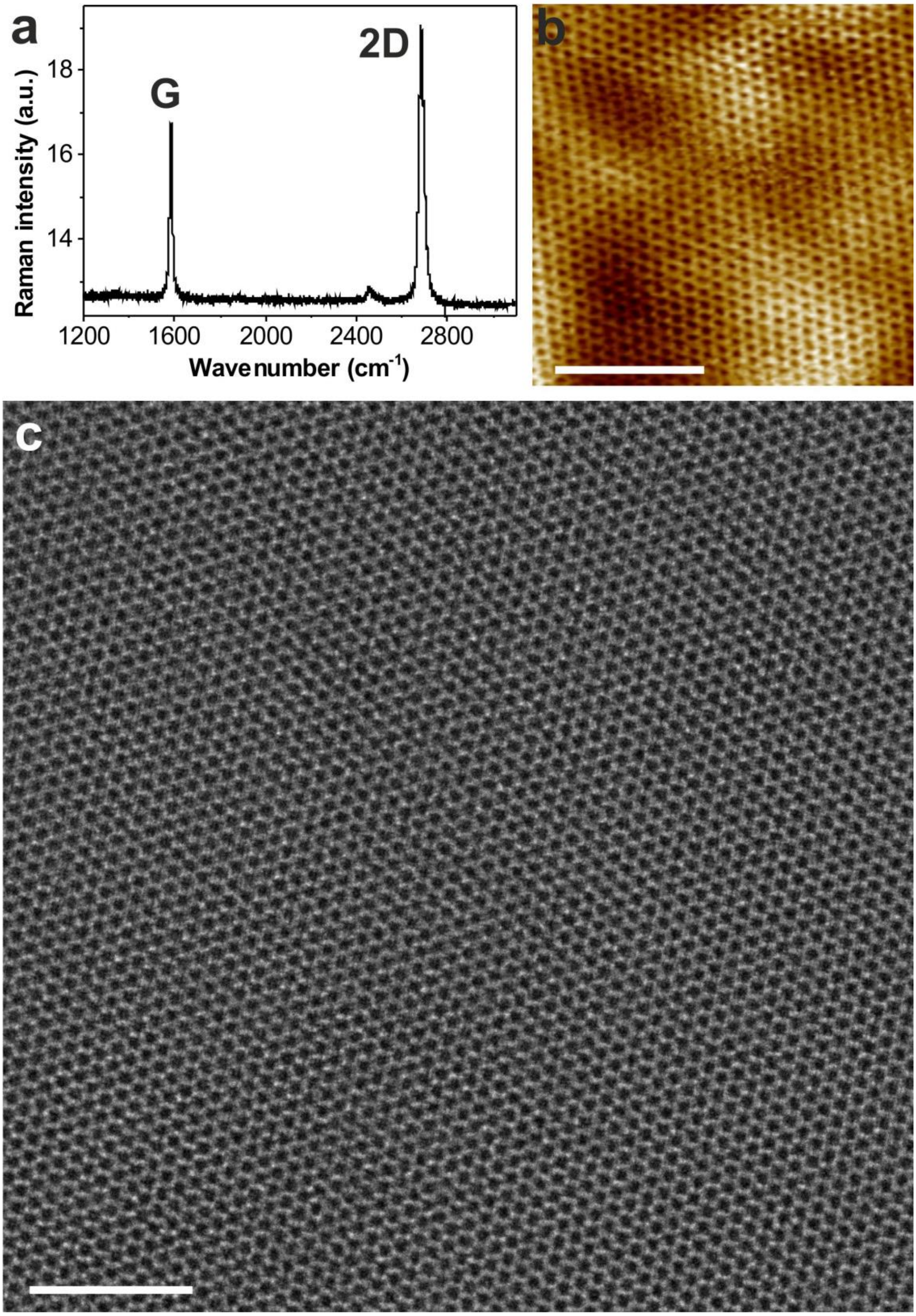

Supplementary Figure 1. Post growth characterization of graphene sheets. a, The Raman spectrum of graphene grown on Pt shows the characteristic G and 2D peaks of single layer graphene. **b,** STM image showing the carbon honeycomb lattice with no

defects. **c** shows an unprocessed high-resolution TEM image of the obtained graphene after transfer, without any additional cleaning steps. The scale bar in **(c)** measures 5 nm.

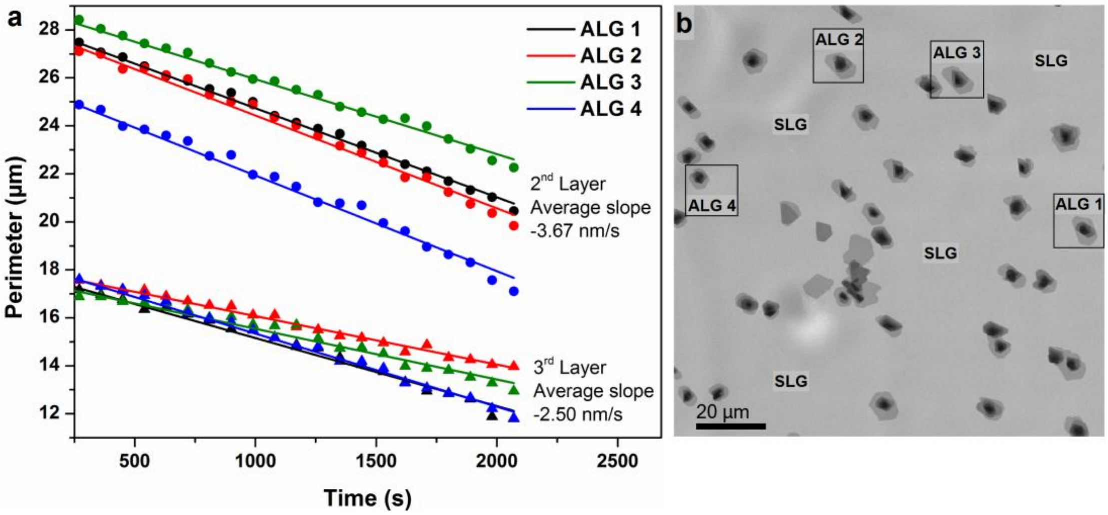

Supplementary Figure 2. Etching rates of 2nd and 3rd layers at 900 °C. a, Plot of the perimeter versus etching time abstracted from 2nd and 3rd adlayers (see Supplementary Movie **M3**). Etching was conducted at 900 °C at 25 Pa H_2. The ratio between the etching rates of the 2nd and 3rd layers are similar to the one discussed in the main text (Average between ALG1-4: 1.5 vs. 1.36 in the main text). **b**, corresponding overview image of ALG on a continuous SLG. The ALG stacks from which the shrinking perimeters were recorded and plotted in **(a)** are indicated by rectangular windows and labelled as ALG1-4. For the analysis, regularly shaped ALG domains were selected in which edges of adlayers were not merged.

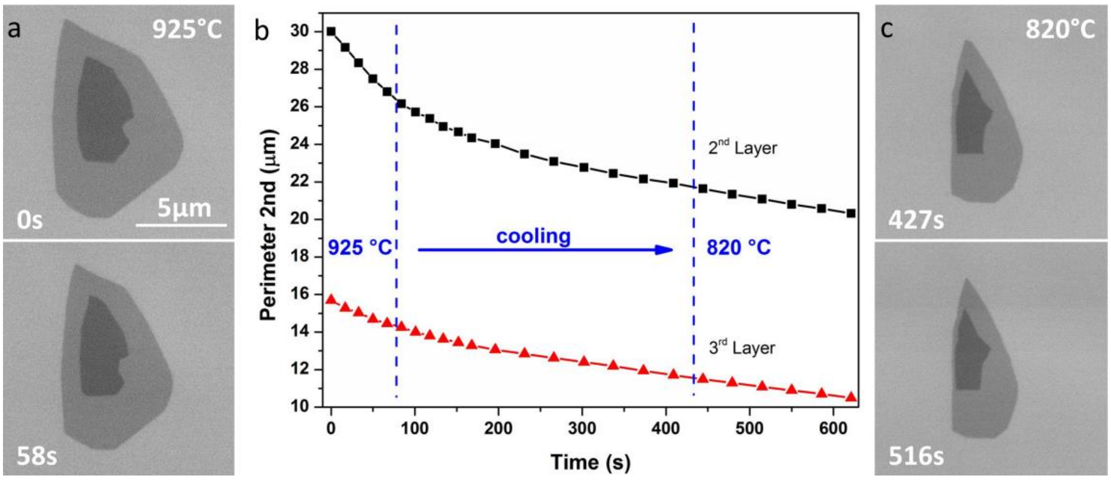

Supplementary Figure 3. Etching of one ALG stack at different temperatures. a, ALG stack showing the 3rd and 2nd layer on a continuous SLG during etching at 925 °C. **b,** Shrinking of the respective perimeters during etching 925 °C, during cooling to 820 °C and at 820 °C. Throughout the observed temperature regime, the etching rate of the 2nd layer was higher than the one of the 3rd layer by a factor of 2.6 at 925°C and 1.3 at 825 °C. **c,** The same ALG stack as shown in **(a)** showing the 3rd and 2nd layer

during etching at 820 °C. Anisotropic shape and etching speed are a consequence of the substrate structure and influence the etching rates. Nevertheless, the 2nd layer etches consistently faster than the 3rd layer.

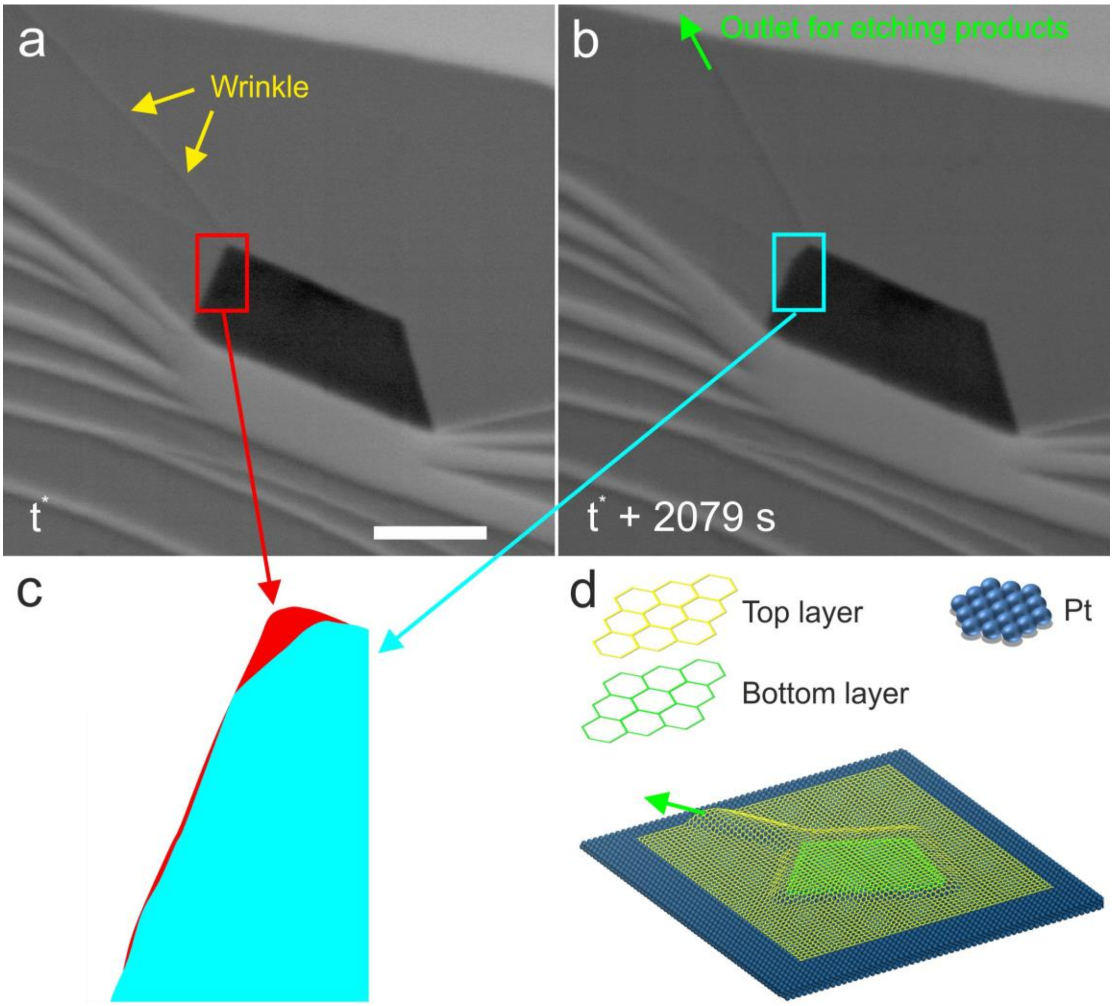

Supplementary Figure 4. Channel formed by a wrinkle. **a, b,** In-situ images showing the etching process of IWC graphene. **c**, Magnified region of **(a)** and **(b)**, showing etching of the ALG underneath the top layer. Etching is enabled through the channel formed by the wrinkle in the top layer. **d**, Schematic showing a wrinkle as outlet for etching products. The scale bar in **(a)** measures 500 nm.

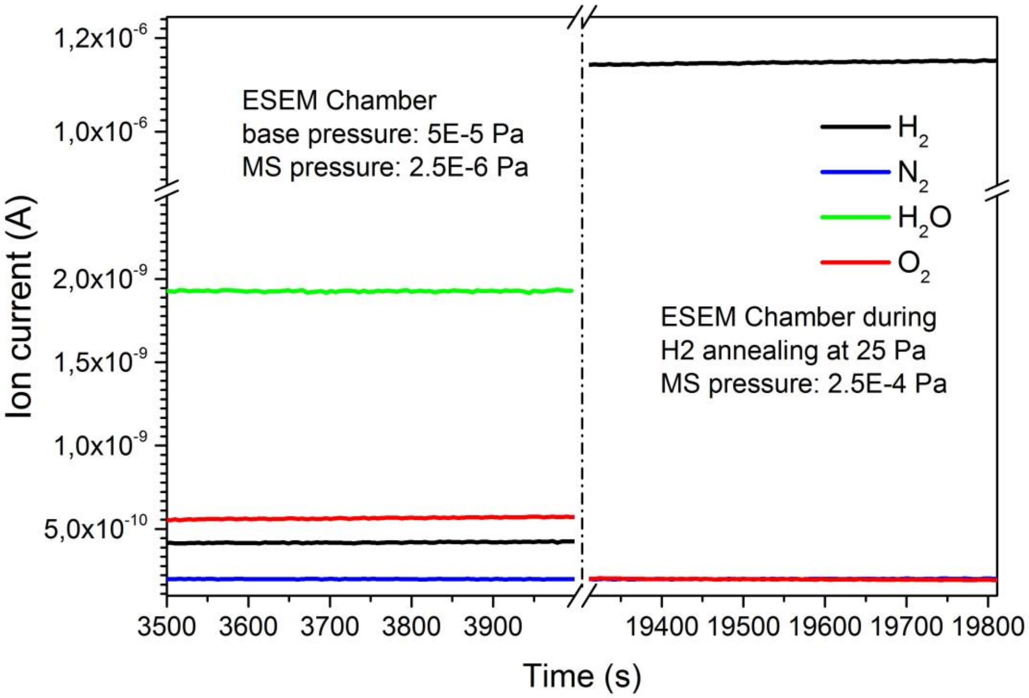

Supplementary Figure 5. Gas composition in the ESEM chamber. Residual gas composition in the chamber of the ESEM at a base pressure of ~5•10^{-5} Pa shows the presence of mainly water, oxygen, hydrogen and nitrogen. The oxygen signal is higher than the nitrogen signal due to contributions from fragmentation of water by electron impact ionization in the MS. Under hydrogen annealing at a chamber pressure of 25 Pa, the gas flow to the MS was restricted by a leak valve to 2.5•10^{-4} Pa. The MS was not calibrated for different pressures and used only to provide qualitative information about the residual gas composition.

Supplementary Note 1. Variation of etching rates

The slower etching rates shown in Supplementary Figure 2 compared to the example discussed in the main text could be a consequence of the full coverage of the Pt grain by a single layer graphene in the case of the adlayer sheets shown in Supplementary Figure 2. Since the Pt catalyst is covered, hydrogen activation by Pt is suppressed and etching relies on thermally activated hydrogen.

Supplementary Note 2. Construction of the polar plots

For the 2D graphene, its growth/etching rate can be simply written as $R(\theta)$, where θ is the angle between one graphene edge from the reference edge which is usually chosen to be the zigzag one. Our previous studies have demonstrated that the growth/etching rate of graphene on Pt (111) substrates has its global minima on zigzag edges, and local minima on armchair edges. In contrast, the growth/etching is fastest when the edge is deviated from the zigzag edge by about 19.1°.[1] Keeping this in mind, we can still qualitatively simulate the growth/etching shape evolution of the graphene flakes in this study, although only the growth rates of zigzag edges can be obtained experimentally. To start with, the growth/etching rates of armchair edges are assumed to be $2.0\times(R_{ZZ\text{-}L}+R_{ZZ\text{-}R})$, where $R_{ZZ\text{-}L}$ and $R_{ZZ\text{-}R}$ are the growth/etching rates of zigzag edges on the left and right sides of the armchair edge. The $R(19.1^{\circ})$ are then given as $1.2\times(R_{ZZ\text{-}R}+R_{AC\text{-}L})$, where $R_{ZZ\text{-}R}$ and $R_{AC\text{-}L}$ are the growth/etching rates of the zigzag and armchair edge on the left and right side of the 19.1° edge. With the above assumptions, the growth/etching rates of all the other edges can be simply obtained by linear interpolation.

Supplementary Note 3. The simulation of shape evolution during etching

The simulated etching of the 1st layer graphene domain is initiated from its final growth shape. A minor modification to the final simulated growth shape of the 2nd graphene layer is made to obtain the initial etching state. For the 3rd layer graphene, the simulated etching is started at an intermediate etching state observed in the experiment.

Supplementary Note 4. Role of SLG wrinkle during etching

Compared to the adlayers on top of the continuous SLG, precipitation layers underneath an existing sheet show a totally different behaviour during hydrogen etching. Because of the high mobility of H on the Pt surface and a high solubility of hydrogen in Pt, H can in principle etch graphene beneath the SLG.[2] Moreover the interplanar spacing between graphene and Pt is close to the interlayer distance of graphite.[3] Normally reactions occurring in nano-sized spaces present novel behaviour due to confinement effects.[4-6] Indeed, recently Yao et. al. found the space between graphene and Pt could serve as a 2D nano-reactor, in which the activation energy of CO oxidation decreased.[7] However, because the etching products cannot easily

diffuse away from the reaction zone, they accumulate until a chemical equilibrium is reached. As a consequence, no substantial etching is observed as long as the precipitated layer is covered by graphene (see Supplementary Fig. 3 a, b). However, if there is the possibility of exchanging species with the environment—for example through a wrinkle in the top sheet—etching can be observed. In this situation, the wrinkle in the top layer acts as a nano-sized exhaust for the etching products (Supplementary Fig. 3). Similarly the graphene wrinkle serves as an inlet for CO to intercalate graphene on Pt.[6] In addition, the evolution behaviour of precipitation layers may give information about edge of top layer.

Supplementary References

1. Ma, T. *et al.* Edge-controlled growth and kinetics of single-crystal graphene domains by chemical vapor deposition. *Proc. Natl. Acad. Sci. U. S. A.* **110**, 20386–20391 (2013).
2. Christmann, K. & Ertl, G. Interaction of hydrogen with Pt(111) - role of atomic steps. *Surf. Sci.* **60**, 365–384 (1976).
3. Sutter, P., Sadowski, J. T. & Sutter, E. Graphene on Pt(111): Growth and substrate interaction. *Phys. Rev. B* **80**, 10 (2009).
4. Thomas, J. M. & Raja, R. Exploiting nanospace for asymmetric catalysis: Confinement of immobilized, single-site chiral catalysts enhances enantioselectivity. *Acc. Chem. Res.* **41**, 708–720 (2008).
5. Smit, B. & Maesen, T. L. M. Towards a molecular understanding of shape selectivity. *Nature* **451**, 671–678 (2008).
6. Mu, R. *et al.* Visualizing Chemical Reactions Confined under Graphene. *Angew. Chem. Int. Ed.* **51**, 4856–4859 (2012).
7. Yao, Y. *et al.* Graphene cover-promoted metal-catalyzed reactions. *Proc. Natl. Acad. Sci. U. S. A.* **111**, 17023–17028 (2014).
